# Effect of Electrospinning Parameters on the Fiber Diameter and Morphology of PLGA Nanofibers

**DOI:** 10.31487/j.dobcr.2021.02.04

**Published:** 2021-05-20

**Authors:** Lohitha Kalluri, Megha Satpathy, Yuanyuan Duan

**Affiliations:** 1 Department of Biomedical Materials Science, University of Mississippi Medical Center, Jackson, Mississippi, USA; 2 Department of Biomedical Materials Science, University of Mississippi Medical Center, Jackson, Mississippi, USA

**Keywords:** Mean fiber diameter, electrospinning, guided tissue regeneration, nanofibers, poly lactic-co-glycolic acid

## Abstract

**Background:**

Poly lactic-co-glycolic acid (PLGA) has been widely investigated for various biomedical applications, such as craniofacial bone regeneration, wound dressing and tissue engineering. Electrospinning is a versatile technology used to produce micro/nanoscale fibers with large specific surface area and high porosity.

**Purpose:**

The aim of the current study is to prepare PLGA nanofibers using electrospinning for guided tissue regeneration/guided bone regeneration applications. The objective of this study is to determine the appropriate electrospinning parameters such as applied voltage, flow rate, spinneret-collector distance and polymer solution concentration for preparation of PLGA fibrous membrane and their effect on the mean fiber diameter of the electrospun fibers.

**Method:**

PLGA pellets were dissolved in Hexafluoroisopropanol (HFIP) in various concentrations overnight using a bench rocker. The resulting PLGA solution was then loaded into a syringe and electrospinning was done by maintaining the other parameters constant. Similarly, various fibrous mats were collected by altering the specific electrospinning parameter inputs such as applied voltage, flow rate and spinneret-collector distance. The morphology of the fibrous mats was characterized using Scanning Electron Microscope. The mean fiber diameter was assessed using ImageJ software and the results were compared using one-way ANOVA.

**Results:**

We obtained bead-free uniform fibers with various tested solution concentrations. One-way ANOVA analysis demonstrated significant variation in mean fiber diameter of the electrospun fibers with altering applied voltage, solution concentration, flow rate and spinneret-collector distance.

**Conclusion:**

The above-mentioned electrospinning parameters and solution concentration influence the mean fiber diameter of electrospun PLGA nanofibers.

## Graphical Abstract

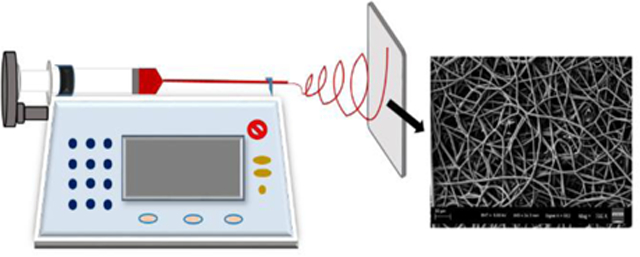

## Introduction

With the advent of nanotechnology, functional polymeric nanofibers have emerged as a promising material in various biomedical applications such as tissue engineering, regenerative medicine, drug delivery, disease modeling and biosensing [1, 2]. In regenerative medicine, 2D and 3D polymeric fibrous scaffolds were designed and fabricated for the regeneration or repair of various tissues such as bone, skin, nerve, heart, blood vessel and musculoskeletal system by tailoring the structural and functional properties including fiber diameter and alignment, porosity, stacking, patterning, surface functional groups, mechanical properties and biodegradability [3, 4]. In the early days, polymeric fibers prepared from natural polymers such as collagen, gelatin, chitosan, silk and alginate were largely investigated for tissue regeneration or repair because of their favourable characteristics as biocompatibility, biodegradability, and solubility in physiological environments [5–18]. However, their applicability was limited by immunogenic properties, unpredictable degradation rate, undefined release kinetics of loaded protein, poor mechanical properties, processing difficulties, cost, availability and the potential risk of transmitting animal-originated pathogens. The limited applicability of natural polymers coupled with the advantages of synthetic polymers such as their formability, mechanical strength tailoring, biocompatibility, flexibility and easily controlled design, etc. has led to the development of synthetic biodegradable polymeric fibers for tissue regeneration applications [19]. Various research groups have investigated the use of polyvinyl alcohol, poly-ε-caprolactone, poly (lactide-co-glycolide) (PLGA) etc. in bone tissue engineering applications [5, 19–24].

Among the synthetic biodegradable polymers, PLGA has been widely used in various biomedical applications such as sutures, drug delivery devices and bone tissue engineering scaffolds, owing to its excellent biocompatibility, controllable biodegradability, tunable degradation rates and mechanical properties. PLGA is the copolymer of L-lactic acid with glycolic acid and its biodegradability can be easily tailored by altering the ratio of lactide: glycolide groups. Also, it is an FDA-approved polymer and can be easily prepared into versatile formulations such as membranes, scaffolds, hydrogels, micro/nanoparticles, and sponges [25, 26]. Recently, PLGA nanofibers were being investigated for use in a wide range of biomedical applications due to their unique properties like the extremely high surface area to weight ratio, low density, high pore volume, small pore size, superior stiffness and tensile strength [23]. Polymeric nanofibers can be prepared by various techniques such as drawing, template-assisted synthesis, self-assembly, phase separation and electrospinning [27, 28]. However, electrospinning has gained wide attention recently owing to its numerous advantages over other techniques, which include versatility, cost-effectiveness, scalability, ability to produce continuous nanofibers with desired patterns, ability to tailor the fiber diameter and its ease of use. Also, the nanofibrous structures obtained with electrospinning have unique properties such as high surface area, high volume-to-mass ratio and inter/intra fibrous porosity [29].

Electrospinning is an electrohydrodynamic process, wherein a polymer liquid droplet is electrified to generate a jet, which is followed by stretching and elongation to generate fibers [30]. Of several factors affecting the electrospinning process, electrospinning parameters such as applied voltage, flow rate of the polymer solution and spinneret-collector distance (S-C distance), and solution parameters like polymer concentration are crucial in affecting the fiber morphology and diameter of the obtained nanofibrous mats [29, 31–35]. Therefore, it is essential to study the effect of these governing parameters on the mean fiber diameter of the PLGA fibrous mats. Also, it is necessary to determine the appropriate parameters and solution concentration to achieve the desired fiber diameter of PLGA nanofibers for intended applications. These parameters vary with the polymer’s physical properties like molecular weight, inherent viscosity, composition etc. To our knowledge, there is little information available regarding the effect of electrospinning parameters and solution concentration to obtain PLGA (75:25) nanofibers for guided tissue regeneration/guided bone regeneration (GTR/GBR) applications.

Thus, the objective of this study was to evaluate the effect of electrospinning parameters and polymer solution concentration on the mean fiber diameter of the electrospun PLGA nanofibers and to determine the appropriate electrospinning parameters and polymer solution concentration for the production of PLGA nanofibers to be used in the GTR/GBR membrane fabrication. The null hypothesis was that there was no significant difference in mean fiber diameter with altered electrospinning parameters and polymer solution concentration.

## Materials and Methodology

**Figure 1:**
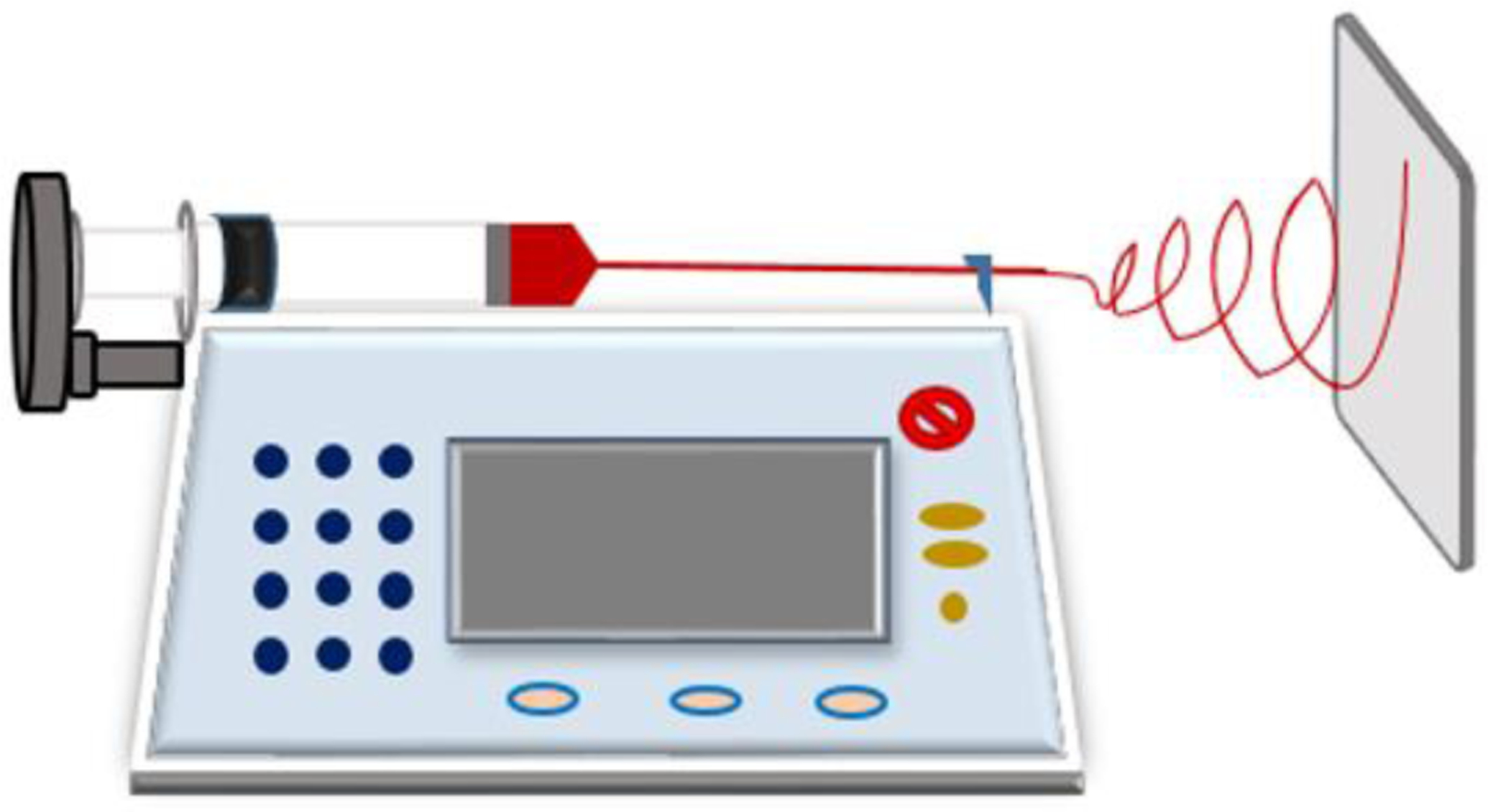
Schematic of electrospinning equipment setup.

Poly (L-lactide-co-glycolide) [75:25; PLGA] was purchased from Corbion (Purac America Inc., Lenexa, USA). 1,1,1,3,3,3-Hexafluoro-2-propanol (HFIP) was purchased from Sigma-Aldrich (Millipore Sigma, St. Louis, USA). All chemicals were used as received without further purification. PLGA (14.5 wt%) was dissolved in HFIP overnight using a bench rocker. The resulting PLGA polymer solution was loaded into a syringe and 18-gauge needle is attached to it. Electrospun nanofibers were fabricated by the electrospinning process using an Inovenso apparatus (Inovenso Inc., MA, USA) which is schematically shown in (Figure 1). The apparatus consists of a propulsion pump, a syringe, a high voltage power supply and a collector. The positive electrode and the negative electrode of the high voltage power supply are connected to the syringe needle and collector plate, respectively. All the samples were collected at the laboratory conditions of 23±2°C temperature and 50% ± 1% relative humidity and left to dry overnight before analysis to allow for the residual solvent evaporation.

To prepare electrospun PLGA fibrous samples at various S-C distances, S-C distance was taken as a variable and samples were collected at 12.5 cm, 15 cm, 17.5 cm and 20 cm, while other parameters were constant as given in (Table 1). Similarly, the applied voltage was taken as a variable for collecting electrospun PLGA fibrous samples at 12 kV, 16 kV and 20 kV, while other parameters were constant as given in (Table 1). Likewise, the flow rate of the polymer solution was taken as a variable for collecting electrospun PLGA fibrous samples at 10μl/min, 15μl/min and 20μl/min, while other parameters were constant as given in (Table 1). In addition to 14.5 wt% PLGA solution, 7 wt% and 11 wt% PLGA solutions were prepared by dissolving PLGA in HFIP overnight using a bench rocker. Electrospun PLGA fibrous samples were prepared from various prepared solution concentrations by maintaining other parameters constant as given in (Table 1). The morphology of all the fibrous mats was characterized using Field-Emission Scanning Electron Microscopy (SEM, Supra 40, Carl Zeiss, Germany). Specimens were gold-coated using a sputter coater (Q150T, Carl Zeiss, Germany) to improve the electrical conductivity, then observed using SEM at a voltage of 5 kV. ImageJ software (ImageJ, National Institute of Health, MD) was used to assess the mean fiber diameter based on the SEM images. At least 30 different positions on each specimen will be measured and the results were compared using one-way ANOVA.

## Results

**Figure 2:**
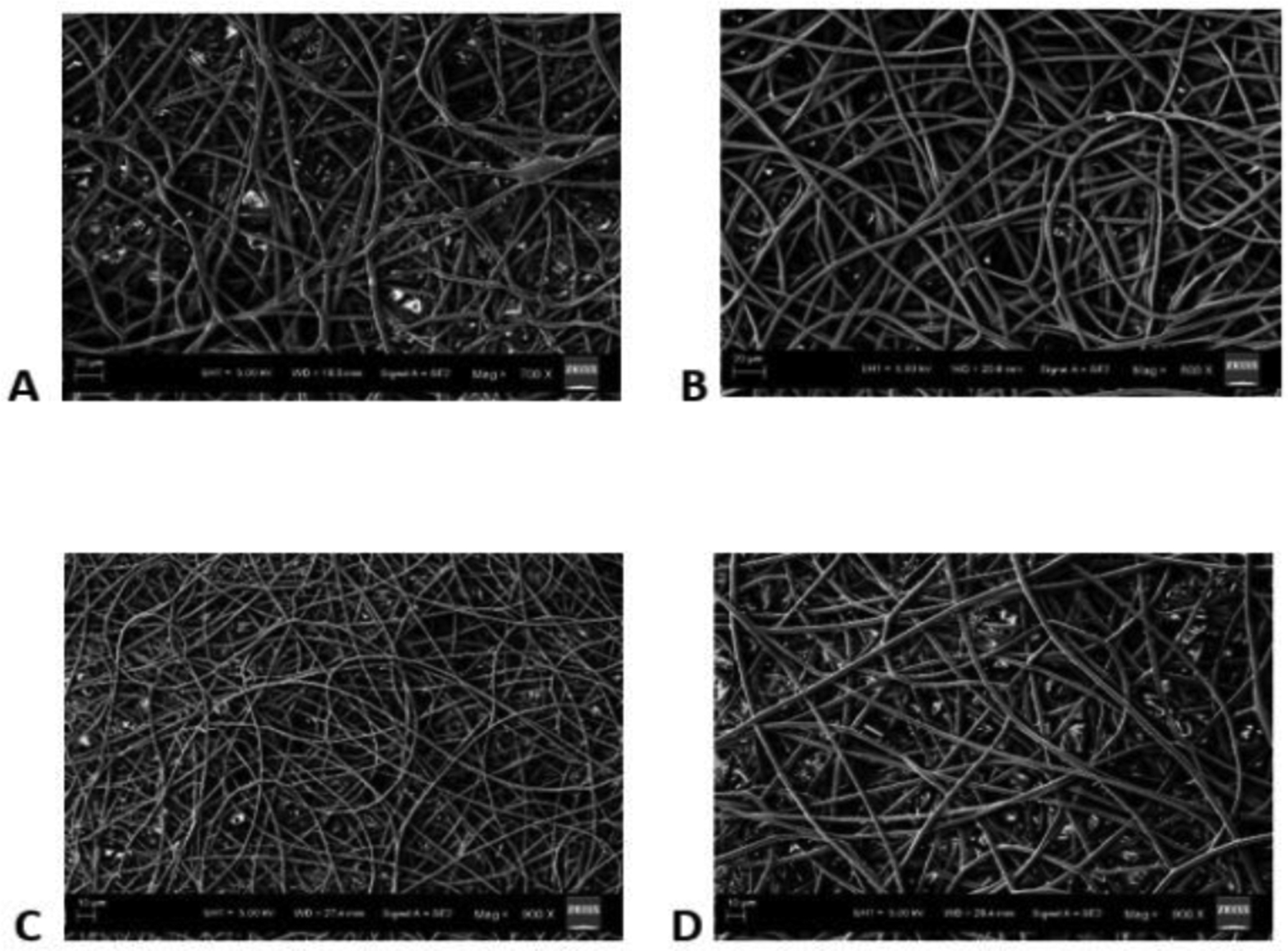
SEM images obtained at S-C distances of **A)** 12.5cm **B)** 15cm **C)** 17.5cm **D)** 20cm.

**Figure 3:**
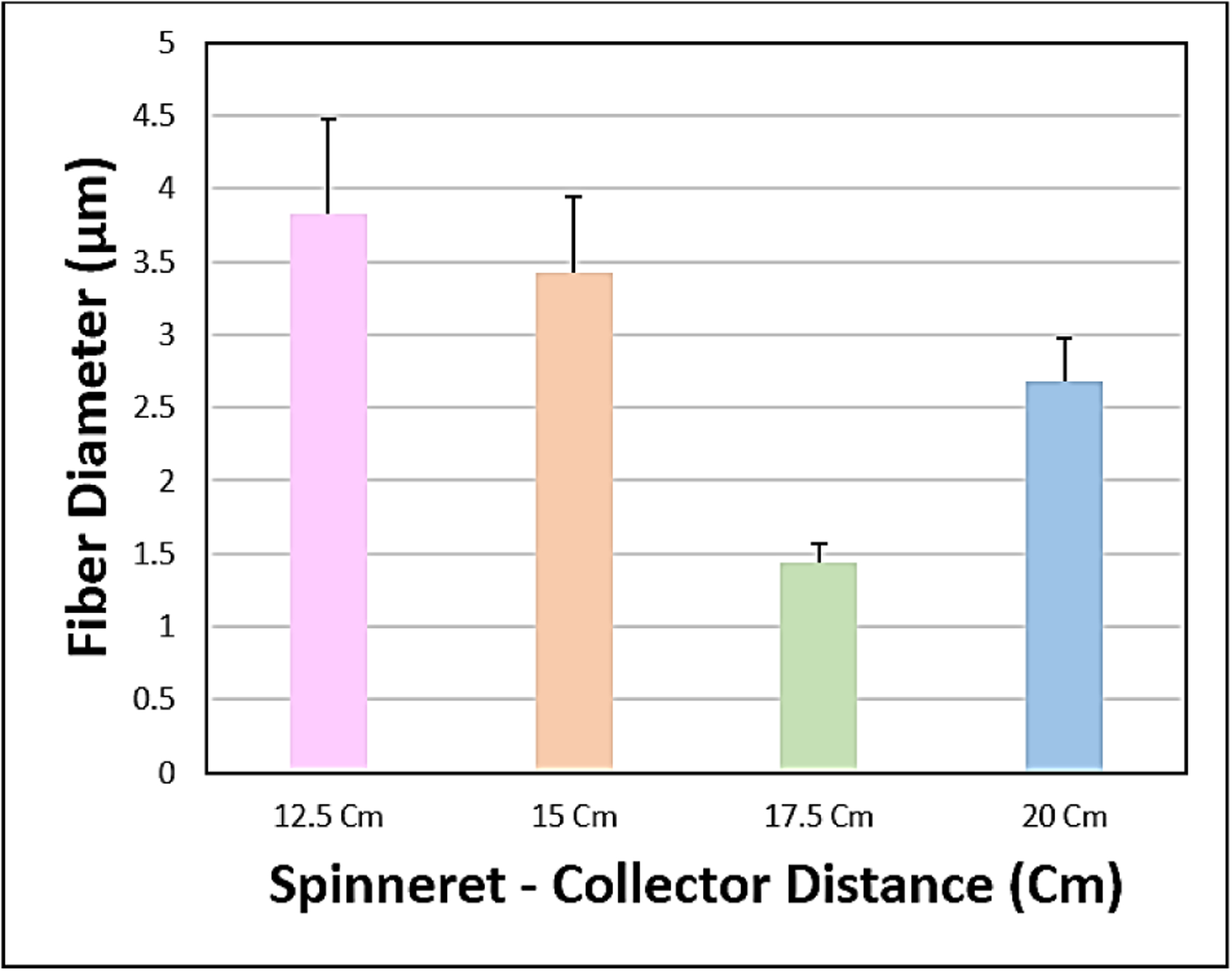
Plot of mean fiber diameter at various S-C distances.

**Figure 4:**
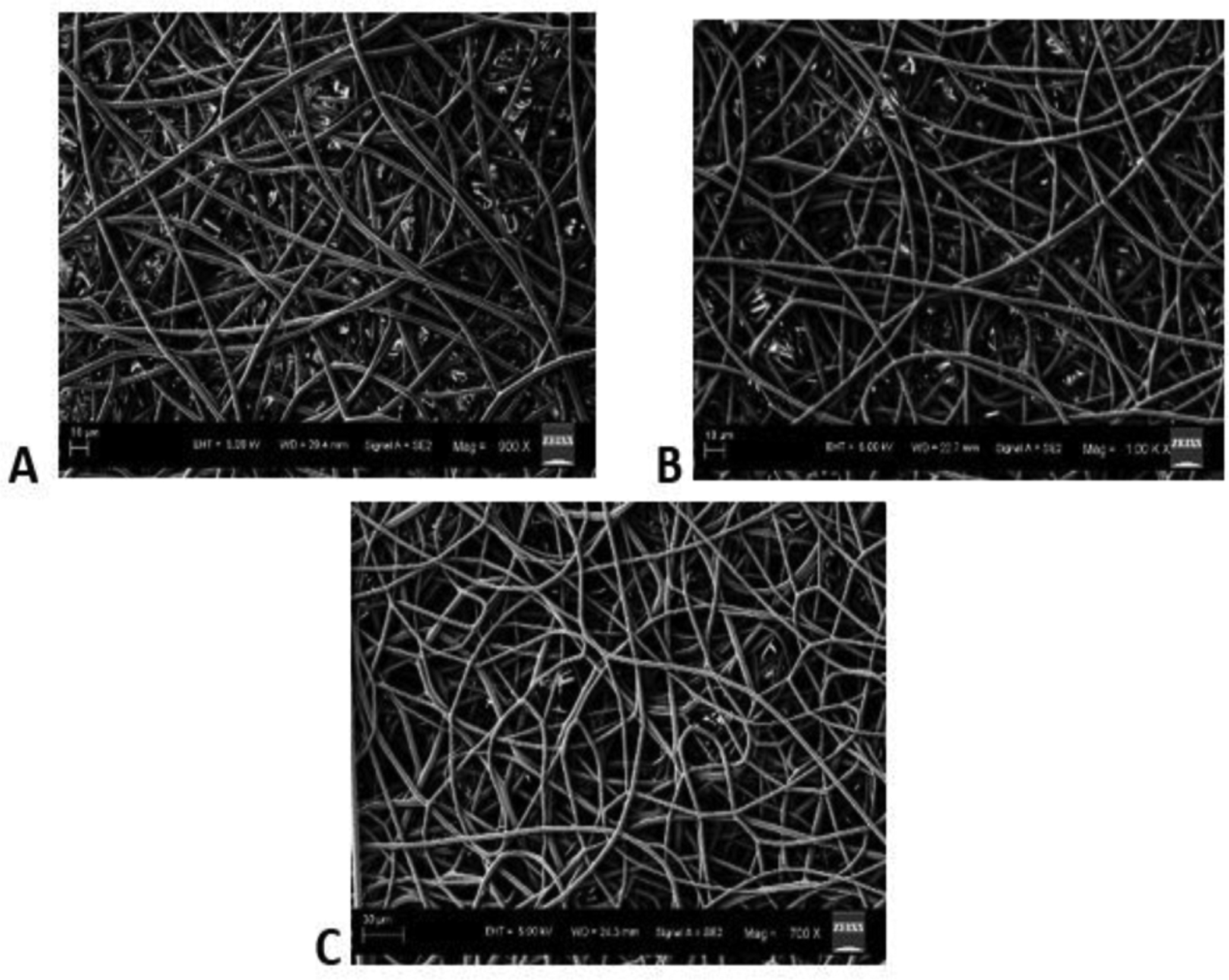
SEM images obtained at applied voltages of **A)** 12 kV **B)** 16 kV **C)** 20 kV.

**Figure 5:**
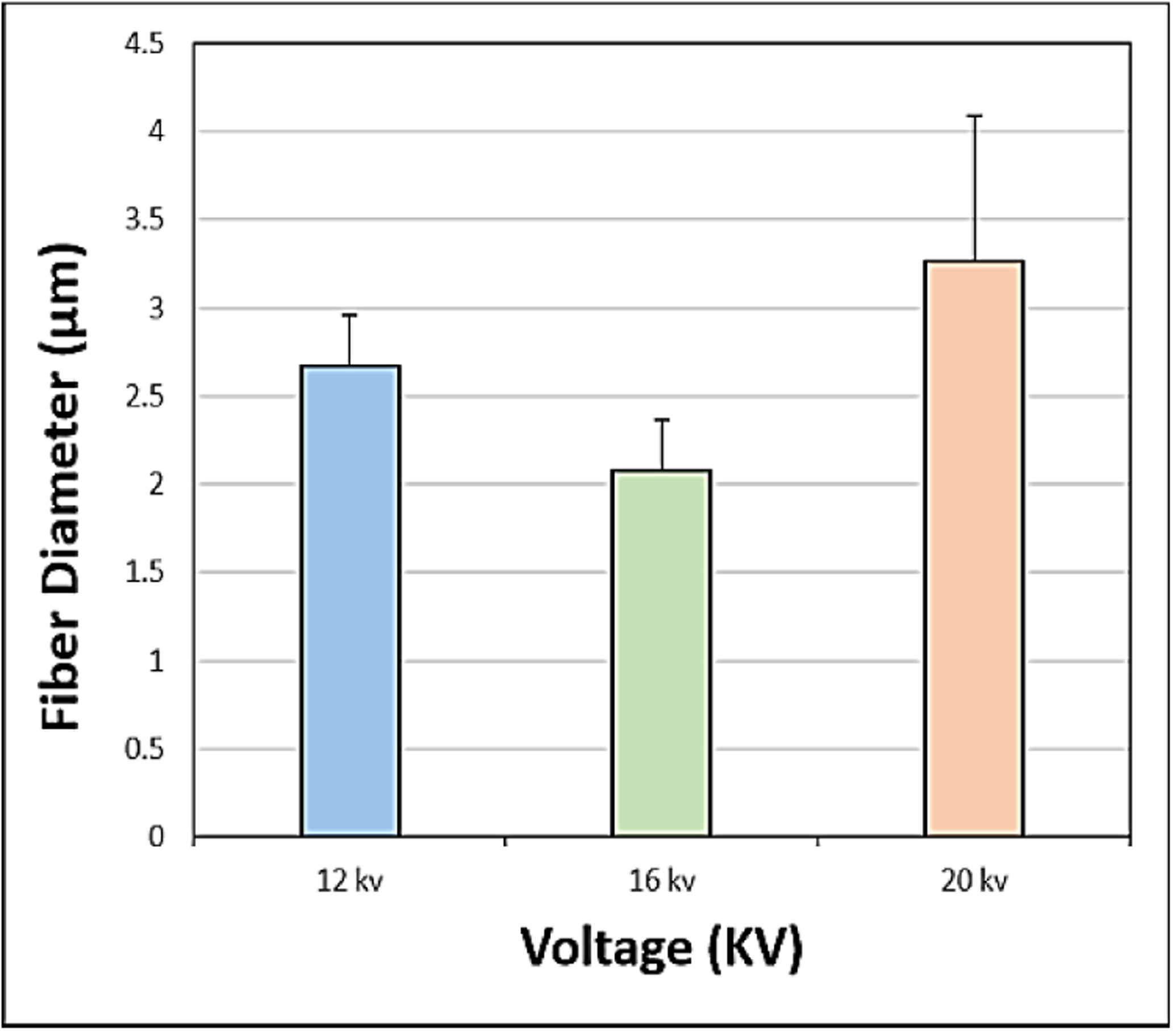
Plot of mean fiber diameter at various applied voltages.

The SEM images obtained at S-C distances of 12.5, 15, 17.5 and 20 cm were depicted in (Figures 2A–2D), respectively, and their corresponding mean fiber diameter values were plotted in (Figure 3). The mean fiber diameter observed at S-C distances of 12.5, 15, 17.5 and 20 cm were 3.8 ± 0.4μm, 3.4 ± 0.2μm, 1.4 ± 0.01μm and 2.7 ± 0.09μm, respectively. One-way ANOVA analysis demonstrated a statistically significant difference (P value = 1.35 E-41) between the mean fiber diameters at different spinneret-collector distances tested. The SEM images obtained with voltages of 12, 16 and 20 kV were depicted in (Figures 4A–4C), respectively and their corresponding mean fiber diameter values were plotted in (Figure 5). The mean fiber diameter observed at applied voltages of 12, 16 and 20 kV were 2.7 ± 0.08μm, 2 ± 0.08μm and 3.2 ± 0.7μm, respectively. One-way ANOVA analysis demonstrated a statistically significant difference (P value = 2.38 E-12) between the mean fiber diameters at different voltages tested.

**Figure 6:**
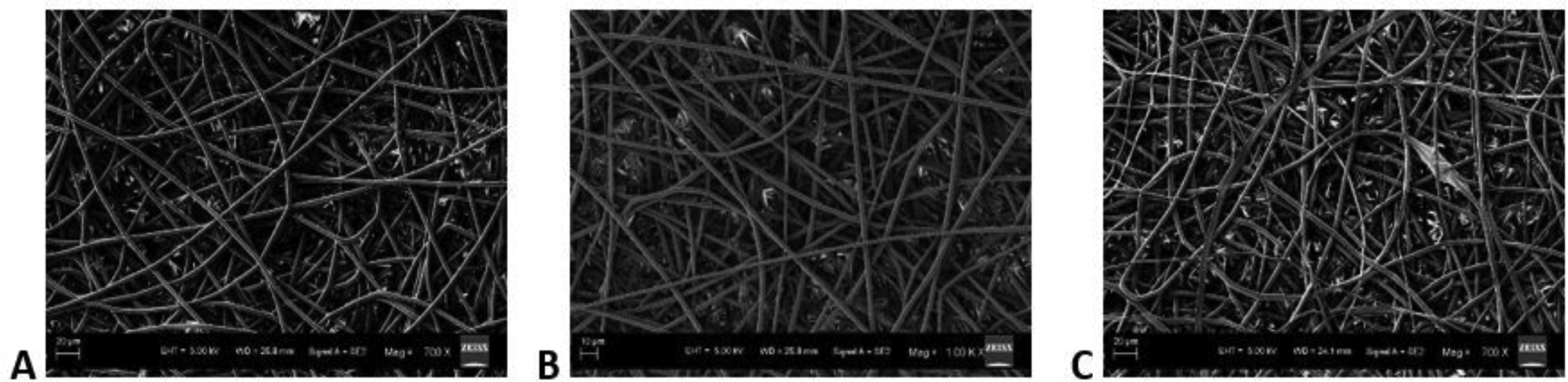
SEM images obtained at polymer solution flow rates of **A)** 10μl/min **B)** 15μl/min **C)** 20μl/min.

**Figure 7:**
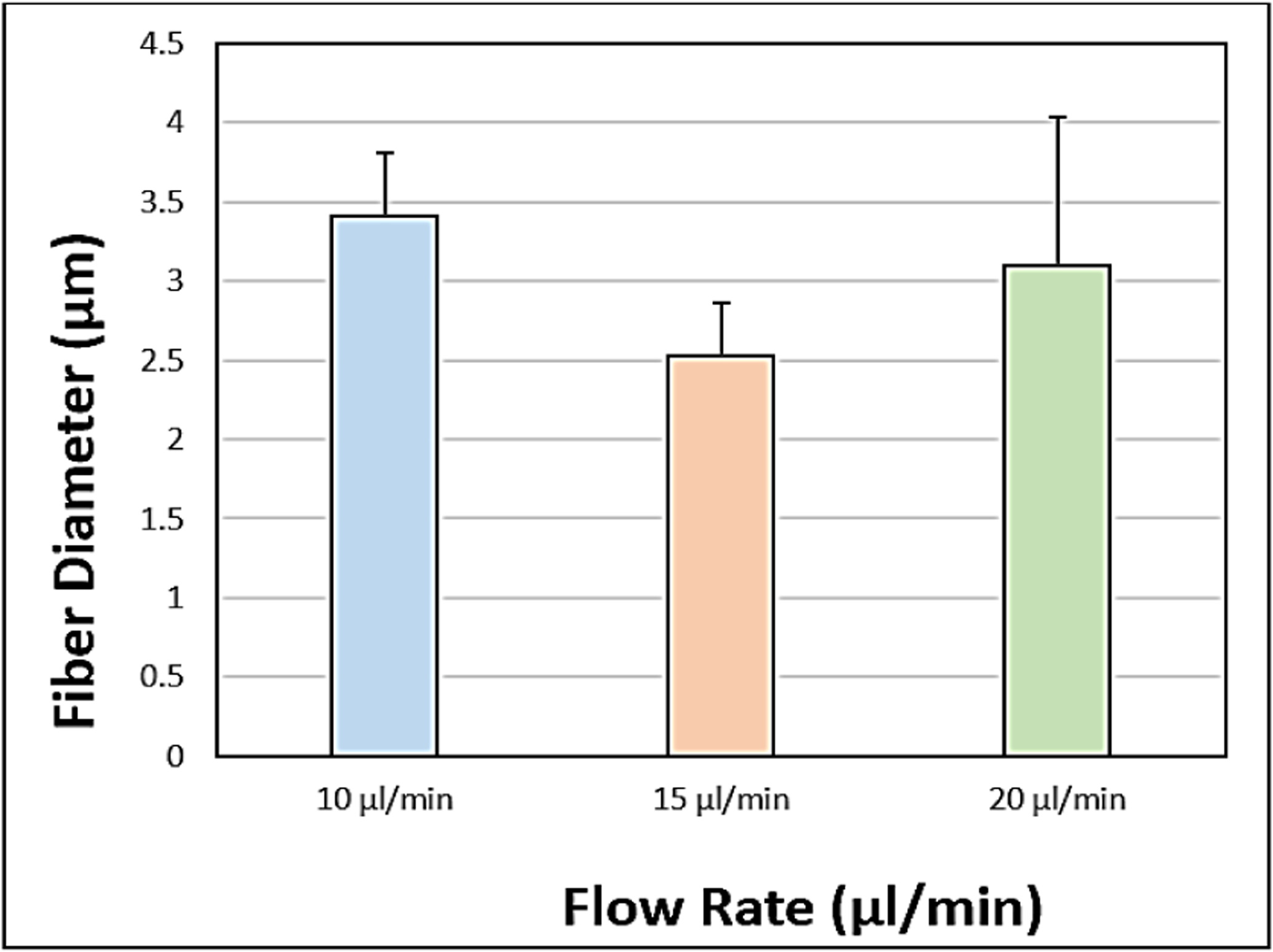
Plot of mean fiber diameter at various polymer solution flow rates.

**Figure 8:**
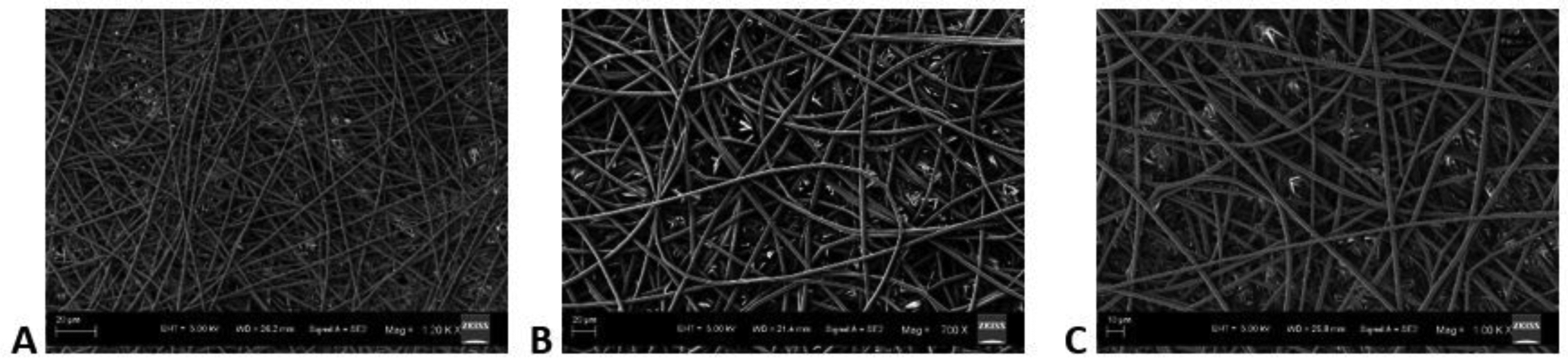
SEM images obtained at polymer solution concentrations of **A)** 7 wt% **B)** 11 wt% **C)** 14.5 wt%.

**Figure 9:**
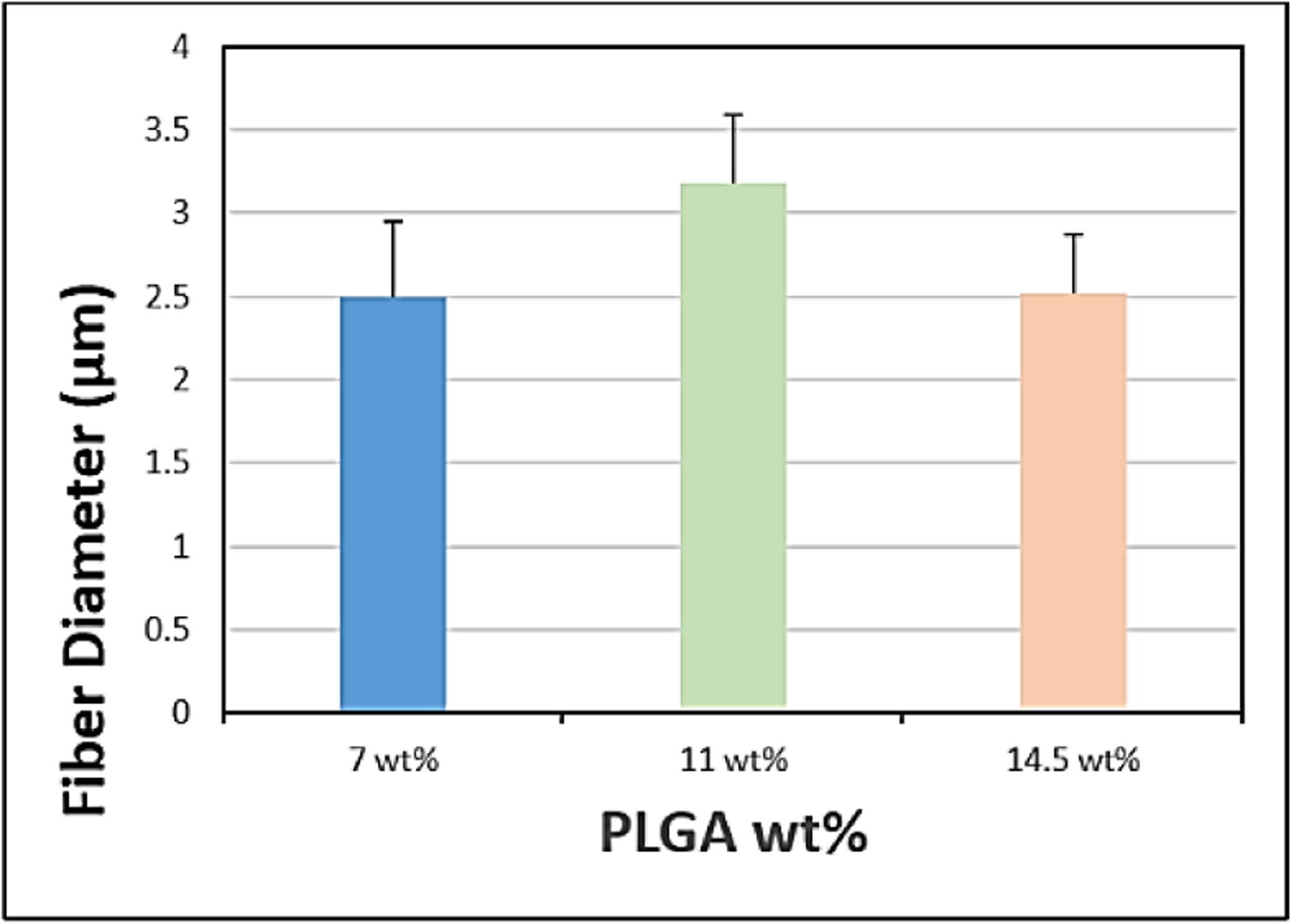
Plot of mean fiber diameter at various polymer solution concentrations.

The SEM images obtained with a flow rate of 10, 15 and 20μl/min were depicted in (Figures 6A–6C), respectively and their corresponding mean fiber diameter values were plotted in (Figure 7). The mean fiber diameter observed at polymer solution flow rates of 10, 15 and 20μl/min were 3.4 ± 0.2μm, 2.5 ± 0.1μm and 3.1 ± 0.9μm, respectively. One-way ANOVA analysis demonstrated a statistically significant difference (P value = 1.19 E-06) between the mean fiber diameters at different tested solution flow rates. The SEM images obtained with PLGA wt% of 7, 11 and 14.5 were depicted in (Figures 8A–8C), respectively and their corresponding mean fiber diameter values were plotted in (Figure 9). The mean fiber diameter observed at polymer solution concentrations of 7, 11 and 14.5 PLGA wt% were 2.5 ± 0.2μm, 3.1 ± 0.1μm and 2.5 ± 0.1μm, respectively. One-way ANOVA analysis demonstrated a statistically significant difference (P value = 8.18 E-10) between the mean fiber diameters at different tested solution concentrations.

## Discussion

SEM micrographs (Figures 2A–2D) demonstrated bead-free uniform fibers at the S-C distances of 15 cm, 17.5 cm and 20 cm. At S-C distance of 12.5 cm, there is a bead formation observed. At spinneret-collector distances of greater than 20 cm, we observed that the fibers were not reaching the collector plate. Thus, the spinneret-collector distances tested were within the range of 12.5 cm to 20 cm. An ideal S-C distance should ensure full extension and solidification of the jets, resulting in the formation of solid fibers and it varies with the polymer system. The morphology and diameter of nanofibers are easily affected by varying S-C distance because it depends on the deposition time, evaporation rate and whipping or instability interval [36]. From (Figure 3), we can observe that there is a decrease in fiber diameter on increasing the spinneret-collector distances from 12 cm to 17.5 cm. This is in consistency with studies conducted by Matabola *et al*. and Wang *et al*. with different polymer systems [36, 37]. However, with a further increase in spinneret-collector distance to 20 cm, there is an increase in mean fiber diameter (Figure 3). Also, with increased S-C distances (17.5 cm and 20 cm), we can observe a narrower fiber diameter distribution. Furthermore, from the observed results, we can conclude that uniform bead-free fibers were obtained at the S-C distance of 17.5 cm.

SEM micrographs (Figures 4A–4C) demonstrated bead-free uniform fibers at various tested voltages. When the voltage was lower than 12 kV, we observed beads and droplet formation on the fibrous mats. There was a frequent clogging of the syringe needle and sparks observed with voltages greater than 20 kV. Thus, the tested voltage range is between 12 kV to 20 kV. The applied voltage directly determines the amount of charges carried by the jet and the magnitude of electrostatic repulsion among the charges, as well as the strength of the interactions between the jet and the external electric field. A higher voltage usually favours the formation of thinner fibers, whereas it may also induce the ejection of more fluid, giving rise to fibers with thicker diameters [33]. From (Figure 5), we can observe that there is a decrease in mean fiber diameter on increasing the voltage from 12 kV to 16 kV. This might be due to the increased amount of charge carried in the jet at higher applied voltages, resulting in the increase of both electrostatic and Coulomb repulsive forces, which in turn might have exerted an increased tensile force on the jet, leading to a reduction in fiber diameter. However, on further increase in voltage to 20 kV from 16 kV, there is an increase in mean fiber diameter and a sudden increase in fiber diameter distribution. This might be attributed to be the induced ejection of more polymer liquid at higher voltages (20 kV). Furthermore, from the observed results, we can conclude that uniform bead-free fibers were obtained at the applied voltage of 16 kV.

SEM micrographs (Figures 6A–6C) demonstrated bead-free uniform fibers at flow rates of 10μl/min and 15μl/min. At 20μl/min flow rate, there is a bead formation and poor fiber uniformity observed. Below 10μl/min, the flow rate was too low to observe the Taylor cone formation. Thus, the flow rates tested were within the range of 10μl/min to 20μl/min. The effect of the flow rate of polymer solution varies with the polymer system tested and the results were inconsistent. Few studies had reported a statistical decrease in the mean fiber diameter with increased flow rate. However, some studies had reported the opposite. Some researchers believed that an increase in the flow rate resulted in an increased ejected volume leading to the increase in fiber diameter [38]. Also, the rapid advancement of the spray solution resulted in a rapid increase in fiber diameter due to incomplete drying of the fibrous mats [29, 39]. From (Figure 7), we can observe that there is a decrease in fiber diameter on increasing the flow rate of solution from 10μl/min to 15μl/min. On further increase in flow rate of solution to 20μl/min, there is an increase in mean fiber diameter and a large increase in fiber diameter distribution. This could be attributed to the increase in the amount of ejected solution with increased flow rate. Furthermore, from the observed results, we can conclude that uniform bead-free fibers were obtained at the flow rate of 15μl/min.

SEM micrographs (Figures 8A–8C) demonstrated bead-free uniform fibers with various tested solution concentrations. When the solution concentration was lower than 7 wt%, we observed poor fiber uniformity of the obtained fibrous mats. The polymer solution was very viscous and there was a frequent clogging of the syringe needle with polymer solution concentrations above 14.5 wt%. Thus, the concentrations of the polymer solutions tested were within the range of 7 wt% PLGA to 14.5 wt% PLGA. From (Figure 9), we can observe that there is an increase in fiber diameter with increasing the polymer solution concentration from 7 wt% to 11 wt%. Increasing the concentration of the polymeric solution results in viscous solution and increased polymer chain entanglements leading to increased fiber diameters [34]. However, on further increase in polymer solution concentration to 14.5 wt%, there is a decrease in mean fiber diameter and the mean fiber diameter of 7 wt% and 14.5 wt% polymer solutions are almost similar. This might be attributed to changes in viscosity and conductivity of polymer solution. Also, at higher concentrations, more uniform fibers with narrower fiber diameter distribution were observed. Furthermore, from the observed results, we can conclude that uniform bead-free fibers were obtained at 14.5 wt% PLGA solution.

Future studies correlating the effect of mechanical properties and physical properties with mean fiber diameters are necessary to determine the applicability of these nanofibers in various biomedical applications. For GTR/GBR membrane applications, adequate mechanical properties are a primary requisite. Thus, optimization of mechanical properties by controlling various electrospinning parameters and mean fiber diameter is necessary to obtain a durable GTR/GBR membrane. Also, characterization of the optimized GTR/GBR membrane to determine the porosity, chemical structure, physical properties and cell culture studies are necessary to determine the applicability of these nanofibers in GTR/GBR membrane applications.

## Conclusion

To conclude, there is a significant variation in the mean fiber diameter with varying the solution concentration and the electrospinning parameters as applied voltage, polymer solution flow rate and S-C distance. Thus, the null hypothesis stating that there was no significant difference in mean fiber diameter with altered electrospinning parameters and polymer solution concentration was rejected. Also, within the limitations of this study, we can conclude that the flow rate of 15μl/min, applied voltage of 16 kV, S-C distance of 17.5 cm and polymer solution concentration of 14.5 wt% PLGA are appropriate for the production of PLGA (75:25) nanofibers to be used in the GTR/GBR membrane fabrication.

## Figures and Tables

**Table 1:** Prepared samples.

Variable	Voltage (kV)	S-C Distance (cm)	Flow Rate(μl/min)	Polymer solution concentration (PLGA wt%)
**S-C Distance**	12	12.5	15	14.5
	12	15	15	14.5
	12	17.5	15	14.5
	12	20	15	14.5
**Applied Voltage**	12	20	15	14.5
	16	20	15	14.5
	20	20	15	14.5
**Flow rate**	20	17.5	10	14.5
	20	17.5	15	14.5
	20	17.5	20	14.5
**Polymer solution concentration**	20	17.5	15	7
	20	17.5	15	11
	20	17.5	15	14.5
